# Ovariotomy

**Published:** 1868-05

**Authors:** 


OVARIOTOMY.—Samuel Cole, M. D., writing from Heidelberg, March 4th, to the *Chicago Medical Journal*, reports in full, a case of ovariotomy, operation having been made in the hospital by Prof. Friederich of the University of Hiedelberg. The tumor removed was a unilocular cyst. Patient healthy and vigorous, not suffering much from the presence of the disease. Patient died at the end of the third week, the following being the anatomical diagnosis: "Peritonitis diffused, encapsuled collections of pus in the vesico and recto-vaginal culs-de-sac. (Douglas'), lobular accumulations in the left lung, fibrinous pneumonia in the right, acute intestinal catarrh and parenchymatous degeneration of the abdominal glands." On the ninth day the "clamp hanging by only a few necrostic shreds, was removed." Spencer Wells clamp was applied to the pedicle, the cyst amputated, and stump cauterized by *ferrum candens*. He says the operation was done in a "masterly manner." The *Chicago Journal* in its "News and Gossip." says: "Prof. Peaslee's last case of ovariotomy died on the fourth day, although apparently doing well until a few hours before death." Hamilton does not like ovariotomy; J. R. Wood, ditto. Too uncertain; it it a kind of surgery you can tell nothing about, etc., etc., as we extract from a private letter from New York. The letter from our Hiedelberg correspondent on this subject will attract attention. So far as present appearances go, the operation is speedily to become much less frequent, if not pass into disuetude."

The operation in Hiedelberg may have been done in a masterly manner, masterly stupid manner, it appears to us. Why leave on the clamp if the pedicle is cauterized? or, why cauterize the pedicle if clamp is to remain? The most "masterly" absurdity ever practiced in making the operation was applying clamp and leaving it outside the incision, dragging upon the pedicle. If in Hiedelberg they still operate in this manner, we think they better suppress their reports and operations. All operations in surgery are "too uncertain." Will they be abandoned? Will capital operations be abandoned because they are uncertain in results? Is it of any use to tell us that Hamilton aud Wood do not like it? not in the least in the world; the impartial record of facts can alone establish or dissuade; ovariotomy does not rest its claims or merits upon being liked or disliked.

